# SPIKENET: An Evidence-Based Therapy for Long COVID

**DOI:** 10.3390/v16060838

**Published:** 2024-05-24

**Authors:** Nila Elumalai, Hussain Hussain, Natarajan Sampath, Nagarajarao Shamaladevi, Rima Hajjar, Brian Zachary Druyan, Amirah B. Rashed, Rajalakshmi Ramamoorthy, Norma S. Kenyon, Arumugam R. Jayakumar, Michael J. Paidas

**Affiliations:** 1 Department of Obstetrics, Gynecology and Reproductive Sciences, University of Miami Miller School of Medicine, Miami, FL 33136, USA; nilanandhie@gmail.com (N.E.); hussainhussainmd77@gmail.com (H.H.); rima.hajjar@jhsmiami.org (R.H.); bzd2@med.miami.edu (B.Z.D.); arashed@med.miami.edu (A.B.R.); rxr1310@med.miami.edu (R.R.); 2 Department of Internal Medicine, HCA Florida Kendall Hospital, Miami, FL 33175, USA; 3 School of Chemical and Biotechnology, SASTRA Deemed University, Thanjavur 613401, Tamil Nadu, India; sams76@gmail.com; 4 Molecular Analytics, Miami, FL 33187, USA; nithumidhu2011@gmail.com; 5 Microbiology & Immunology and Biomedical Engineering, Diabetes Research Institute, University of Miami, Miami, FL 33136, USA; nkenyon@med.miami.edu; 6 Department of Biochemistry and Molecular Biology, University of Miami Miller School of Medicine, Miami, FL 33136, USA

**Keywords:** COVID-19, multiorgan dysfunction, molecular mechanism, long COVID, SPIKENET, therapy

## Abstract

The COVID-19 pandemic has been one of the most impactful events in our lifetime, caused by severe acute respiratory syndrome coronavirus-2 (SARS-CoV-2). Multiple SARS-CoV-2 variants were reported globally, and a wide range of symptoms existed. Individuals who contract COVID-19 continue to suffer for a long time, known as long COVID or post-acute sequelae of COVID-19 (PASC). While COVID-19 vaccines were widely deployed, both unvaccinated and vaccinated individuals experienced long-term complications. To date, there are no treatments to eradicate long COVID. We recently conceived a new approach to treat COVID in which a 15-amino-acid synthetic peptide (SPIKENET, SPK) is targeted to the ACE2 receptor binding domain of SARS-CoV-2, which prevents the virus from attaching to the host. We also found that SPK precludes the binding of spike glycoproteins with the receptor carcinoembryonic antigen-related cell adhesion molecule 1 (CEACAM1) of a coronavirus, murine hepatitis virus-1 (MHV-1), and with all SARS-CoV-2 variants. Further, SPK reversed the development of severe inflammation, oxidative stress, tissue edema, and animal death post-MHV-1 infection in mice. SPK also protects against multiple organ damage in acute and long-term post-MHV-1 infection. Our findings collectively suggest a potential therapeutic benefit of SPK for treating COVID-19.

## 1. Introduction

The COVID-19 pandemic has sparked an unparalleled global health crisis, with documented cases surpassing 774 million and a devastating toll of over 7 million lives lost worldwide [1]. Despite the decrease in mortality rates attributed to the development of effective vaccines and therapeutics, the threat of SARS-CoV-2 persists, underscoring its formidable impact on global public health. Notably, the virus’s high mutation rate has given rise to numerous variants, with recent strains such as XBB.1.5, XBB.1.16, EG.5, BA.2.86, JN.1, XBB, XBB.1.9.1, and XBB.2.3 contributing to the highest hospitalization rates since the emergence of the Omicron variant in 2022 [2]. Of increasing concern are the 10–20% of individuals diagnosed with COVID-19 who experience symptoms persisting for months to years beyond their initial infection. The comprehensive understanding of COVID-19’s long-term health impact remains a focal point of active investigation, pivotal to international public health efforts to develop effective treatment and management strategies moving forward [3].

While SARS-CoV-2 is typically characterized as a respiratory viral infection, acknowledging its multi-organ involvement and broad range of associated symptoms provides a more comprehensive understanding of COVID-19. In addition to affecting the respiratory system, SARS-CoV-2 has been detected in the brain, liver, pancreas, skin, and heart, underscoring its impact on multiple organ systems [1,2,3]. Notably, our murine hepatitis virus-1 (MHV-1) mouse model demonstrates multi-organ involvement and exhibits most symptoms associated with COVID-19 in humans. Precisely, the MHV-1 mouse model displays symptoms including drowsiness, reduced mobility, weight loss, early-stage diarrhea, ruffled fur, altered hind limb posture, mild-to-moderate dyspnea, fatigue, and tremors and progresses to a moribund state culminating in death [4].

In the MHV-1 mouse model, numerous pathologic changes occur across multiple organ systems during acute infection [4]. In the brain, observed pathologic changes include congested blood vessels, perivascular cavitation, pericellular halos, vacuolation of neuropils, darkly stained nuclei, pyknotic nuclei amid associated vacuolation of the neuropil, and acute eosinophilic necrosis. Cardiac tissue samples display evidence of interstitial edema, vascular congestion, and infiltration of red blood cells between degenerative myocardial fibers. Renal changes consist of tubular epithelial cell degeneration, proximal and distal tubular necrosis, interstitial tissue hemorrhage, and vacuolation of renal tubules. Similarly, liver pathology indicates hepatocyte degeneration, severe periportal hepatocellular necrosis characterized by pyknotic nuclei, congestion, ballooned hepatocytes, vacuolation, piecemeal necrosis, ground glass hepatocytes with voluminous, abundant, granular cytoplasm, as well as peripheral cytoplasmic clearing and central nuclei. During acute infection, apoptotic (acidophil) bodies, abundant inflammatory cells, condensation and dark cytoplasm staining, fatty changes, binucleated hepatocytes, and activated Kupfer cells are also evident.

Furthermore, examination of lung tissue samples obtained from the MHV-1 mouse model reveals severe inflammation, proteinaceous debris filling the alveolar spaces with fibrillar to granular eosinophilic protein strands, the presence of hemosiderin-laden macrophages, peribronchiolar interstitial infiltration, bronchiole epithelial cell necrosis, necrotic cell debris within alveolar lumens, alveolar exudation, hyaline membrane formation, alveolar hemorrhage, and interstitial edema [4]. Notably, the clinical symptoms observed in our MHV-1 mouse model closely mirror the symptomatology identified in patients with the SARS-CoV-2 variants mentioned above [5,6,7].

Furthermore, our MHV-1 mouse model also displayed long-term sequelae affecting multiple organ systems during the 12 months following the initial injection with MHV-1, resembling aspects of long COVID-19 disease observed in humans. Brain tissue samples exhibited severely congested blood vessels, perivascular cavitation, pericellular halos, vacuolation of neuropils, pyknotic nuclei, eosinophilic necrosis, necrotic neurons with fragmented nuclei, and vacuolation. Cardiac tissue samples demonstrated severe interstitial edema, vascular congestion, myocyte necrosis, inflammation, and apoptotic bodies. The kidneys showed tubular epithelial cell degenerative changes, interstitial tissue hemorrhage, vacuolation of renal tubules, inflammation of the renal parenchyma, severe tubular necrosis, and infiltration of macrophages and lymphocytes. Liver samples revealed congestion, ballooned hepatocytes, vacuolation, piecemeal necrosis, and occasional ground glass hepatocytes with voluminous, abundant, granular cytoplasm, central nuclei, and activated Kupffer cells. Additionally, lymphocyte infiltration in sinusoidal spaces, multifocal hepatic necrosis both in the periportal area and near the terminal hepatic veins, increased number of portal veins associated with luminal severe dilatation, activated Kupffer cells with large cytoplasm containing necrotic debris, eosinophilic bodies, mitotic cells, balloon-like liver cells, mild inflammation of the lobular lymphocytic and portal tract, mild hydropic degeneration of liver parenchymal cells, and lipofuscin pigment were also noted.

Regarding pulmonary samples, observations included proteinaceous debris filling the alveolar spaces with fibrillar to granular eosinophilic protein strands, the presence of hemosiderin-laden macrophages, peribronchiolar interstitial infiltration, bronchiole epithelial cell necrosis, necrotic cell debris within alveolar lumens, alveolar exudation, hyaline membrane formation, alveolar hemorrhage, interstitial edema, bronchioles with thickened airway walls due to fibrotic remodeling (likely attributed to excessive deposition of collagen bundles), bronchioles containing a large intraluminal mucous plug, bronchioles with increased numbers of goblet cells in the epithelial lining, and bronchiole walls with increased numbers of inflammatory cells in the lungs [8].

Understanding the pathophysiology of COVID-19 disease progression beyond acute infection and delineating resultant organ dysfunction are imperative for the development of effective treatments. Today, identifying a pharmaceutical agent capable of halting disease progression is paramount. We propose that our innovative peptide, SPIKENET, represents a promising therapeutic agent capable of effectively impeding disease progression and averting subsequent long-term complications.

## 2. Current Treatment Strategy

Several antiviral medications have been approved by the Food and Drug Administration (FDA) to mitigate disease severity and decrease the risk of hospitalization in mild-to-moderate cases of COVID-19. These medications include Paxlovid, Remdesivir, and Molnupiravir [9]. Additionally, the FDA issued emergency authorization to prescribe immunomodulatory medications, such as baricitinib and tocilizumab, for patients hospitalized due to SARS-CoV-2 infection [9].

Based on past experiences combating similar viruses like SARS-CoV-1 and Middle Eastern Respiratory Syndrome (MERS), pharmaceutical industries accelerated the development of vaccines targeting SARS-CoV-2. The vaccines developed during the COVID-19 pandemic are widely recognized for their role in reducing SARS-CoV-2-related deaths. However, despite progress in drug development and the introduction of effective vaccines, a subset of individuals continues experiencing symptoms long after the resolution of their initial infection, contributing to various long-term health complications. Such cases are commonly referred to as “long COVID” by the public. The most prevalent long-term sequelae encompass disrupted sleep patterns, compromised bone health, fertility issues, musculoskeletal problems, cardiovascular complications, exacerbation of diabetes, liver dysfunction, pulmonary issues, kidney dysfunction, and neurological as well as psychiatric complications [3,10]. Remarkably, despite the duration of the pandemic, data elucidating the precise nature and trajectory of SARS-CoV-2 infection, along with its long-term consequences, need to be revised, and this necessitates further investigation.

## 3. SPIKENET Invention and Therapeutic Applications

Diverse histological alterations were discovered during our examination of MHV-1 infection within the murine integument, probing into the acute, long COVID, and SPK treatments (SPK did not display any changes in the control group, which was identical to the healthy group; [8]). Currently, no treatments are available to eliminate post-acute SARS-CoV-2 complications (PASC) or long COVID resulting from mild-to-moderate SARS-CoV-2 infection. Our laboratory has developed potential therapeutic targets and strategies to address long COVID. Drawing from the molecular structure of the SARS-CoV-2 spike glycoprotein-1 (S1) and its interaction with the angiotensin-converting enzyme-2 (ACE2) receptor for cell entry [11], we designed a peptide, termed SPIKENET (SPK), comprising 15 amino acids. Employing computational programs such as computational docking and molecular dynamic studies, we focused on the spike glycoprotein, crucial for viral entry into the cell via the ACE2 receptor [11,12]. This synthetic peptide is engineered to specifically bind to the S1 glycoprotein, impeding SARS-CoV-2 entry into the host cell. Notably, this approach has demonstrated efficacy against various SARS-CoV-2 variants (SPK is patent-protected by the University of Miami Miller School of Medicine Office of Technology Transfer—Inventors, Arumugam R. Jayakumar and Michael J. Paidas).

**Figure 1:**
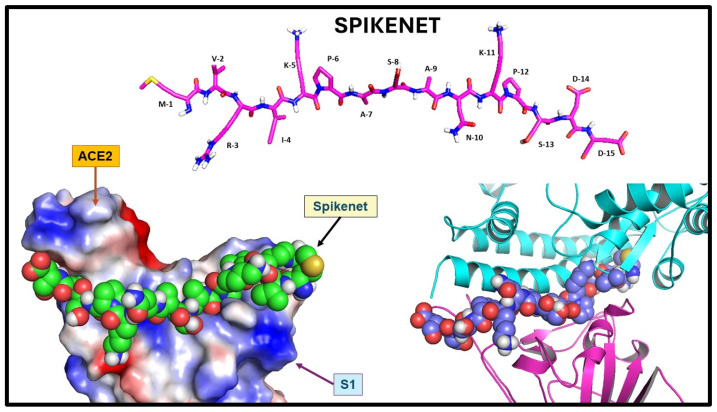
SPIKENET structure and binding affinity to the ACE2 binding domain of the SARS-CoV-2 spike glycoprotein. Structure of SPIKENET, a 15-amino-acid synthetic peptide targeted to the ACE2 binding domain of the SARS-CoV-2 spike glycoprotein (top image). The computational protein docking approach shows particular SPIKENET binding affinity to the ACE2 and CEACAM1 binding domains of the SARS-CoV-2 and MHV-1 spike glycoproteins (S1), respectively (bottom images) [13].

**Figure 2:**
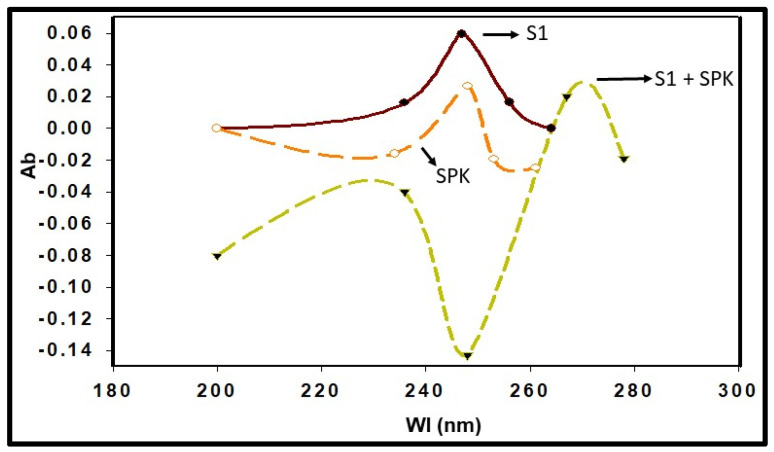
Spectroscopic analysis shows highly specific SPIKENET (SPK) binding affinity to the ACE2 binding domain of the SARS-CoV-2 spike glycoprotein (S1)—Ab, absorbance; Wl, wavelength [13].

We synthesized a series of candidate peptides to target the S1 glycoprotein of the SARS-CoV-2 virus. Among these peptides, SPK exhibited the highest efficacy in binding to the ACE2 receptor. Our investigation revealed a specific binding affinity of SPK to the ACE2 binding domain of the SRAS-CoV-2 S1. The computational protein docking approach shows particular SPIKENET binding affinity to the ACE2 and CEACAM1 binding domains of the SARS-CoV-2 and MHV-1 spike glycoproteins (S1), respectively (Figure 1) [13]. Further, our spectroscopic analysis demonstrates particular SPK binding affinity to the ACE2 binding domain of the S1 (Figure 2). The peptide, composed of 15 amino acids, showed a theoretical mass of 1628.9 Daltons, with an observed mass of 1628.4 Daltons, indicating high purity at 98.7%. Sigma/Aldrich synthesized the peptide (Saint Louis, MO, USA).

For administration, the pharmaceutical formulation of SPIKENET can be delivered intravenously, transmucosal, orally, subcutaneously, or intramuscularly, with the recommended dosing frequency of twice daily for both human subjects and experimental animals. The product is supplied as a white lyophilized powder in vials containing 10 mg, devoid of preservatives, and exhibits chemical stability for 72 h post-reconstitution when stored between −20 and −40 °C. The lyophilized powder can be stored at temperatures ranging from −20 to −80 °C, with an alternative storage option at −20 °C for 3–6 months and longer durations (6–48 months) at −80 °C. Shelf-life information is provided on the product information sheet, and administration beyond the indicated expiration date on the package and vial is not recommended. Reconstitution of the peptide should adhere to aseptic techniques to ensure product sterility, and any unused solution should be discarded as per the Environmental Protection Agency guidelines.

A molecular docking investigation explored the interactions between the receptor binding domain (RBD) of SARS-CoV-2 and SPIKENET, our proprietary synthesized peptide. This study sought to improve our understanding of the non-bonded interactions, peptide conformational preferences over the human ACE2 binding site within the RBD domain of the COVID-19 virus, and the binding affinity of the peptide. Following the docking simulation, ten distinct conformations of the SPIKENET peptide were generated. Among these conformations, the model exhibiting the highest binding affinity (−156.2 kcal/mol) was selected for subsequent detailed binding analysis.

**Figure 3:**
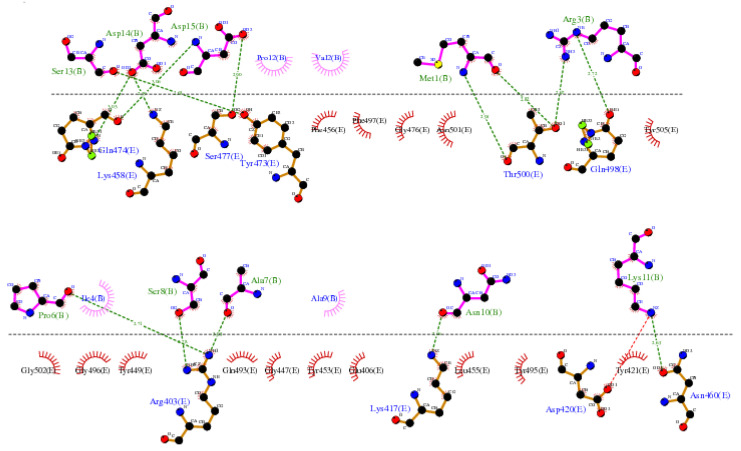
The LIGPLOT diagram of the protein–peptide interaction between the RBD and SPIKENET. The pink SPIKENET peptide residues are shown on the top, and the yellow amino acid residues of the RBD from SARS-CoV-2—two are shown at the bottom. The SPIKENET peptide consists of 15 amino acid residues, 14 of which are shown in non-bonded interactions with the RBD of SARS-CoV-2, proving that SPIKENET has a significant binding affinity with the spike glycoprotein-1. These findings strongly suggest that SPIKENET is a potent competitive inhibitor of S1 [13].

The LIGPLOT diagram illustrating the protein-peptide interaction between the RBD and SPIKENET is depicted in Figure 3. The SPIKENET peptide residues, highlighted in pink, are presented at the top, while the amino acid residues of the RBD from SARS-CoV-2 are depicted in yellow at the bottom. Comprising 15 amino acid residues, the SPIKENET peptide engages in non-bonded interactions with 14 residues within the RBD domain of SARS-CoV-2, underscoring its significant binding affinity with the COVID-19 Spike protein [13]. Notably, our analysis of structural data reveals a striking similarity between the residues involved in SPIKENET peptide binding and those necessary for binding with human ACE2 receptors, facilitating viral infection in humans.

Further examination of the complex structure of RBD-SPIKENET interaction exhibits strong electrostatic intermolecular interactions (within the range of from 2.59 to 3.06 Å distances) between specific residues of the RBD domain, including Gln-474, Lys-458, Ser-477, Tyr-473, Thr-500, Gln-498, Arg-403, Lys-417, Asp-420, and Asn-460, and residues of the SPIKENET peptide, namely Ser-13, Asp-14, Asp-15, Met-1, Arg-3, Pro-6, Ser-8, Ala-7, Asn-10, and Lys-11. Additionally, hydrophobic interactions are observed between residues Phe-456, Phe-497, Gly-476, Asn-501, Tyr-505, Gly-502, Gly-496, Tyr-449, Gln-493, Gly-447, Tyr-453, Glu-406, Leu-455, Tyr-495, and Tyr-421 and residues Pro-12, Val-2, Ile-4, and Ala-9 from the SPIKENET peptide, further enhancing peptide stability at the ACE2 binding site of the RBD [13].

Based on our computational docking and spectroscopic analyses, we embarked on pilot studies to evaluate the potential protective effects of our peptide against COVID-19. Two distinct animal models of COVID-19 were selected for this investigation. The first model utilized MHV-1-induced mice to simulate COVID-19, while the second model employed humanized mice infected with SARS-CoV-2 (K18-hACE2 transgenic mouse), developed in collaboration with Dr. Bridget Barker from Northern Arizona University. The MHV-1 mouse model was previously developed in our laboratory under BSL-2 containment and documented in several publications [14,15,16,17,18].

After developing these animal models, pilot studies were conducted to assess the impact of SPIKENET on animal survival. These studies yielded compelling results indicative of significant protection against infection with SARS-CoV-2. Detailed data from these pilot studies are provided below.

Given that the MHV-1 virus utilizes carcinoembryonic antigen-related cell adhesion molecule-1 (CEACAM1) as its host receptor [18], our initial investigation aimed to ascertain whether our peptide exhibited binding affinity with the MHV-1 spike protein. Then, to ensure broad specificity towards various forms of spike proteins, including the different SARS-CoV-2 variants that have emerged, we engineered our peptide to target these proteins specifically. To validate this, we first confirmed the interaction of our peptide with the MHV-1 N protein’s N-terminal Domain (NTD).

**Figure 4:**
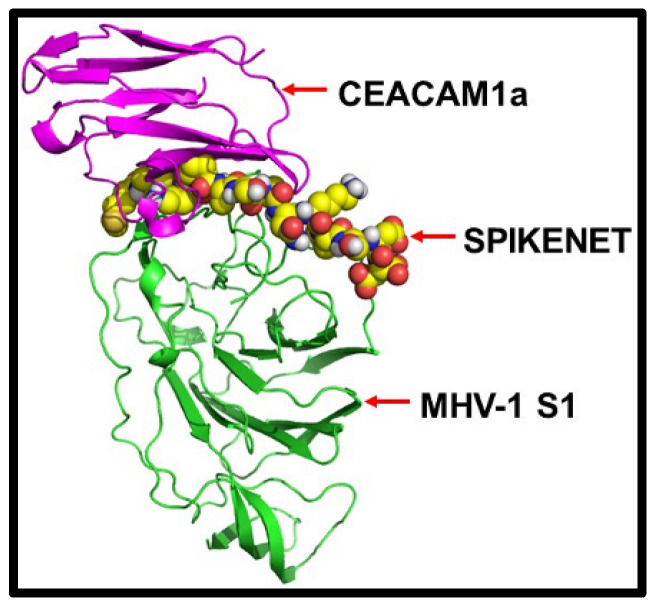
The protein docking approach shows SPIKENET binding affinity to the CEACAM1a binding domain of the MHV-1 spike protein [13].

**Figure 5:**
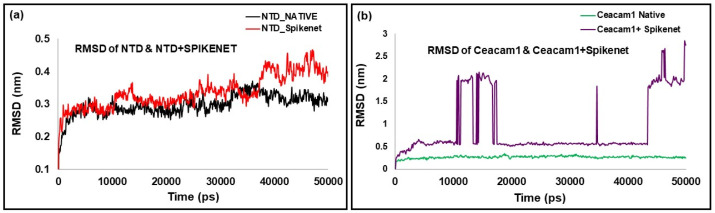
SPIKENET binding affinity to the CEACAM1a (CCM) binding domain of the MHV-1-NTD with molecular dynamic studies. The RMSD of both complexes with their respective native proteins are shown in (**a**,**b**). The structures showed complete equilibration in the system when comparing the RMSD of both NTD and NTD + SPK (**a**) after 50 ns dynamic simulation. The SPK peptide was well stabilized and had a high affinity at the CCM binding location of NTD. However, the RMSD analysis of CCM and CCM+ SPK structures after the 50 ns dynamic simulation exhibited more flexibility at the NTD binding site of CCM than native CCM (**b**), suggesting the SPK peptide detachment and displacement over the CCM [13].

Using molecular docking and molecular dynamics simulations, we rigorously examined the binding interactions between our peptide, MHV-1 NTD, and CEACAM1. Our investigations found a robust binding affinity of SPIKENET with the CEACAM1 binding domain of the MHV-1 NTD, mirroring the affinity observed with SARS-CoV-2 S1. Alternatively, this binding was not observed with ACE2 or CEACAM1, as evidenced by both docking and molecular dynamics analyses, as illustrated in Figure 4 and Figure 5. Briefly, confirmatory computer molecular dynamic studies show a RMSD of both complexes with their respective native proteins (Figure 5). The structures showed complete equilibration in the system when comparing the RMSD of both NTD and NTD + SPK (a) after 50 ns dynamic simulation. The SPK peptide was well stabilized and had a high affinity at the CCM binding location of NTD. However, the RMSD analysis of CCM and CCM+ SPK structures after the 50 ns dynamic simulation exhibited more flexibility at the NTD binding site of CCM than native CCM (b), suggesting the SPK peptide detachment and displacement over the CCM.

While it is unclear how our peptide targets any forms of spike proteins, including any variants, the highly hydrophobic binding environments of receptor binding domains (RBD) may favor the high binding affinity of SPK to any variants. We earlier demonstrated multiple sequence alignment (MSA) of human RBDs from SARS-CoV-2 and N-terminal domain (NTD) from MHV-1, confirming that both these domains share less than 12% sequence similarity, which suggests that these structures are dissimilar. Moreover, the structural alignment of the RBDs and NTDs of viral spike proteins showed a root mean square deviation (RMSD) of 17.4 Å, which also strongly supports the structural dissimilarity. Still, they hold the β-sheets in their core structure. Interestingly, the investigation of both human receptor binding sites by the creation of an electrostatic surface diagram revealed that the structures are dissimilar, but receptor binding sites of both structures have almost similar, highly hydrophobic binding environments. Hence, both these domains can strongly bind with SPIKENET.

While it is unclear how SPIKENET has a high binding affinity with S1 proteins of both SARS-CoV-2 and MHV-1, we examined multiple sequence alignments (MSA) of human RBDs from SARS-CoV-2 and NTD from MHV-1 receptor binding sites by creating an electrostatic surface diagram. While we found that the structures are different, the RBDs of both structures have similar, highly hydrophobic binding environments, resulting in a remarkably high binding affinity with SPIKENET. 

## 4. SPIKENET Protective Effect against Animal Mortality

We conducted a comprehensive assessment to determine the efficacy of our peptide in mitigating animal mortality, weight loss, clinical symptoms, and pathological alterations associated with SARS-CoV-2 infection. In addition to evaluating these significant clinical parameters and survival rates, we investigated critical pathogenic events speculated to contribute to the progression of SARS-CoV-2 infection.

**Figure 6:**
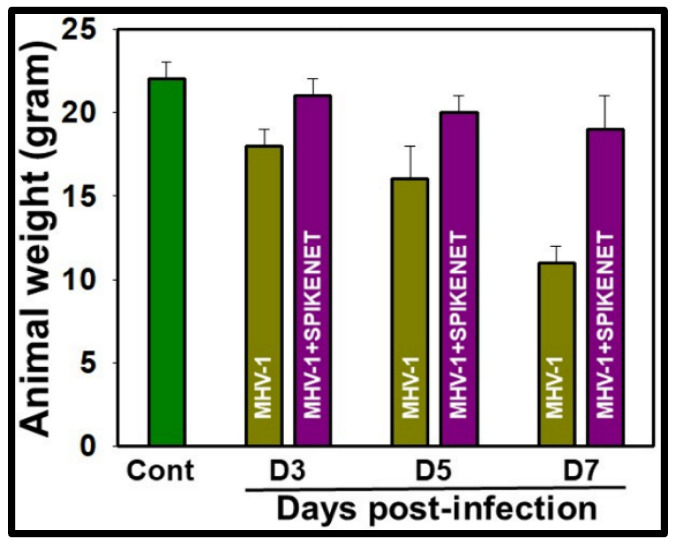
MHV-1-inoculated mice lost 20–40% of body weight over days 3–7. Treatment of MHV-1-inoculated mice with SPIKENET (3 doses, on days 2, 4, and 6 post-MHV-1 injection with 5 mg/kg) reversed such loss. These findings correlated well with the animal survival post-SPIKENET treatment in SARS-CoV-2-infected K18-hACE2 transgenic mice (*n* = 10). Error bars represent mean ± SEM [13].

**Figure 7:**
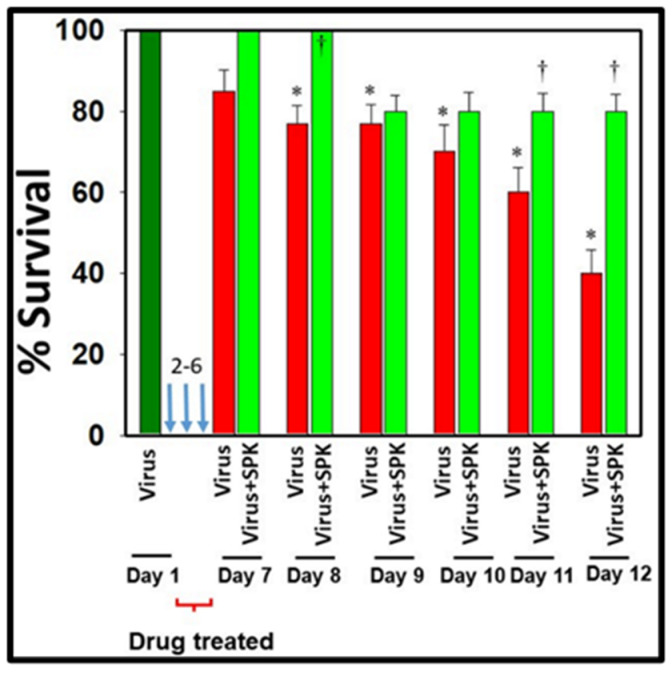
SPIKENET treatment reduces the death rate in MHV-1-infected mice. Female A/J mice were inoculated with MHV-1 intranasally. SPIKENET (5 mg/kg b.wt.) was injected subcutaneously when mice showed sickness (i.e., 2 days after MHV-1 exposure). SPIKENET was injected 2 times every alternate day. SPIKENET diminished the animals’ deaths. ANOVA, *n* = 5 for control, *n* = 16 for virus alone, and *n* = 7 for MHV1 + SPK group. * = *p* < 0.05 versus control; † = *p* < 0.05 verses MHV-1 infected mice, Error bars represent mean ± SEM [13].

These analyses encompassed the examination of body weight fluctuations (Figure 6), multi-organ pathological changes, oxidative/nitrative stress, development of multi-organ edema, and alterations in the expression of water channel proteins aquaporins 1, 4, 5, and 8 in the MHV-1 mice model of COVID-19. Our findings suggest substantial protection against these pathological changes alongside improved animal survival (Figure 7) when MHV-1 mice were treated with SPIKENET (5 mg/kg) three times, administered every alternate day beginning from the onset of sickness, i.e., day two post-injection [13].

Furthermore, we investigated the long-term sequelae of COVID-19 in these mice through 12 months post-infection. Our observations unveiled irreversible pathological alterations in multiple organs, with pronounced impairments evident in the brain, lungs, and heart compared to the liver and kidneys. Interestingly, the virus persisted in all organs tested during acute infection and in the long-term post-infection phase. Treatment of MHV-1-infected mice with SPIKENET significantly attenuated disease progression and the pathological changes observed during long-term infection. These results further emphasize COVID-19’s role in provoking enduring, irreversible alterations primarily affecting the brain, lungs, and heart. Additionally, our results highlight the therapeutic potential of SPIKENET in mitigating both acute and long-term pathological consequences associated with SARS-CoV-2 infection.

Corroborating our findings in the MHV-1 mouse model, we recently documented SARS-CoV-2 in human brain samples obtained from a prematurely born neonate delivered at our facility to a mother infected with SARS-CoV-2 [19]. This notable case involved a pregnant individual presenting to our facility at 27 weeks gestation with severe respiratory symptoms and pneumonia. Upon initial examination, she tested positive for SARS-CoV-2 via RT-PCR. The mother was then admitted to the intensive care unit (ICU) and received treatment for pneumonia and multisystem disease while undergoing continuous fetal monitoring.

Due to the deteriorating maternal clinical condition, an emergent Cesarean section was performed at 32 weeks of gestation. Immediately following delivery, the newborn exhibited seizure-like activity and respiratory distress. At 24 h of life, COVID-19 IgG and combined IgG/IgM/IgA reactivity to a recombinant derivative of the SARS-CoV-2 spike protein were detected in the infant’s serum, accompanied by markedly elevated serum inflammatory markers and cytokine levels. MRI conducted on day 2 of life revealed a left germinal matrix and left ventricular hemorrhage. Follow-up imaging at 10 weeks of life indicated resolution of bleeds, yet severe parenchymal atrophy was observed. The infant was discharged home after three months with a diagnosis of seizure disorder and acquired growth failure, with abnormal neurological examination persisting at the 12-month follow-up evaluation [19].

Tragically, at 13 months of age, the infant experienced asystolic cardiac arrest and was transported to the emergency department, where resuscitation attempts were unsuccessful. Autopsy findings revealed a significant reduction in brain weight, enlarged ventricles, reactive gliosis, and neuronal death throughout the cerebral, cerebellar, and brainstem white matter [19].

**Figure 8:**
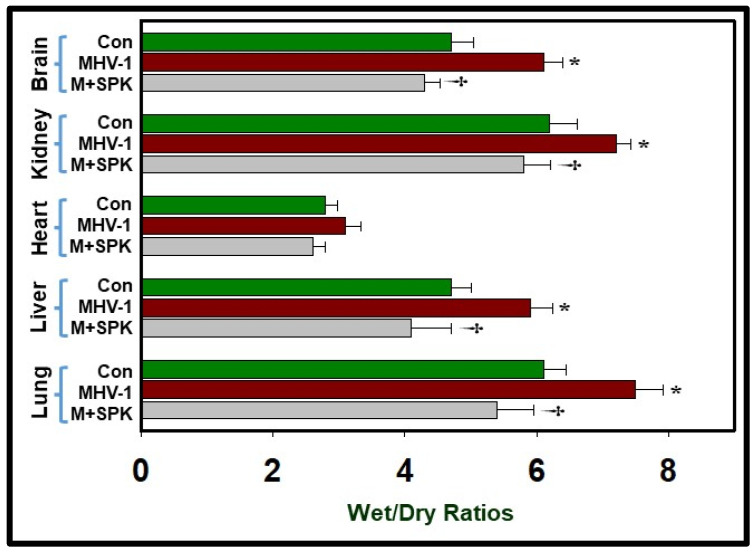
Elevated edema was observed in MHV-1-infected brain, lung, liver, kidney, and heart, as compared to control, which was similar to patients with COVID-19. Treatment of MHV-1-infected mice with SPK (5 mg/kg; 3 injections from 2 to 6 days) showed edema levels comparable to control levels on day 7. ANOVA, *n* = 5 for control, *n* = 16 for virus alone, and *n* = 7 for MHV1 + SPK group. * = *p* < 0.05 versus control; † = *p* < 0.05 verses MHV-1 infected mice, Error bars represent mean ± SEM [13].

We detected the presence of both spike protein (S1) in the brain and the nucleocapsid protein, which indicates viral presence after SARS-CoV-2 infection [19]. Furthermore, we observed a blood–brain barrier breakdown and reduced levels of ACE2 in the infant’s brain. The discovery of SPIKENET’s protective effect against the development of acute edema in MHV-1-infected mice, coupled with insights from SARS-CoV-2 infection in humans, underscores a possible link between generalized edema and the progression of COVID-19 disease. We also noted increased edema with varying severity in multiple organs, including the lung, skin, liver, brain, kidney, and heart [13,19,20,21]. Treatment of MHV-1-inoculated mice with SPIKENET yielded edema levels comparable to controls on day 7 post-infection (Figure 8). Unlike SPIKENET, the administration of another small molecular peptide (VRIKPGTANKPSED) to MHV-1-inoculated mice did not impact animal survival, consistent with its lack of binding affinity with S1 or ACE2/CEACAM-1 [13]. Taken together, these findings suggest that the development of edema in various organs may represent a seminal event in SARS-CoV-2 infection, and our newly developed peptide, SPIKENET, which effectively prevents S1 binding with ACE2 or CEACAM-1, holds promise as a therapeutic agent targeting SARS-CoV-2 infection and decreasing risk of long-term adverse effects on overall health and wellbeing.

In addition to its role in preventing generalized edema, SPIKENET’s efficacy against acute oxidative stress is equally noteworthy. Administration of 5 mg/kg of SPIKENET to MHV-1-infected mice reversed physiologic changes attributed to oxidative stress. While redox system alterations have been implicated in the pathophysiology of SARS-CoV-2 infection [22], our current understanding of the impact of oxidative stress and its ramifications on pathophysiological changes during infection remains limited.

**Figure 9:**
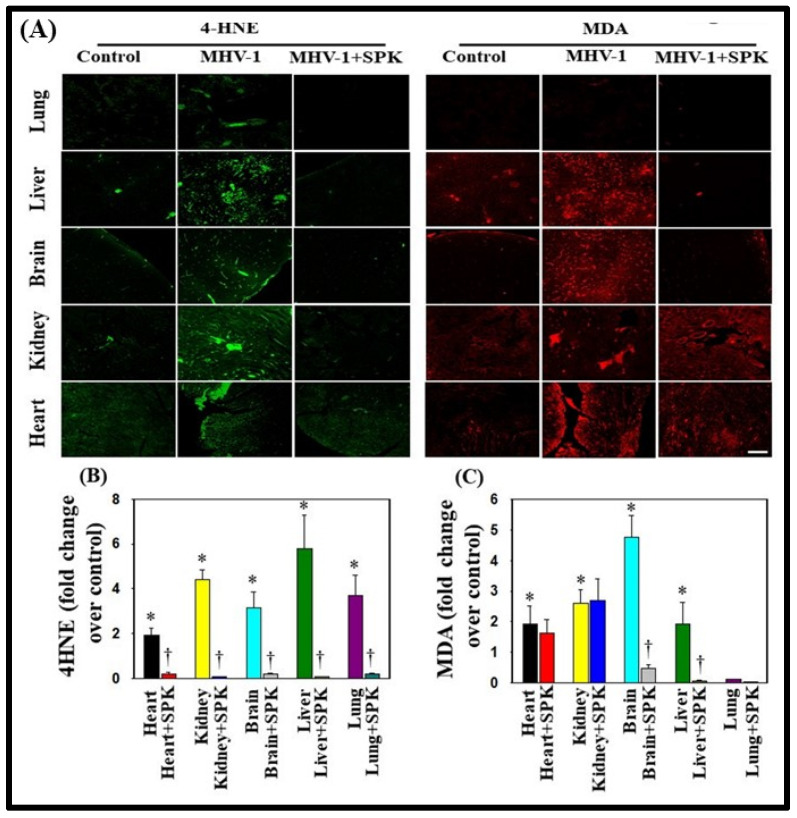
Oxidative stress post-MHV-1 infection in mice. (**A**) Representative immunofluorescence images from four individual animals show increased levels of 4-hydroxynonenol (4-HNE) and malondialdehyde (MDA) in the lung, liver, kidney, brain, and heart. Treatment of MHV-1-inoculated mice with SPK (5 mg/kg) prevented such an increase. (**B**,**C**) Quantitation of 4-HNE and MDA levels with and without SPK post-MHV-1 infection. ANOVA, *n* = 4. * = *p* < 0.05 versus control; † = *p* < 0.05 verses MHV-1-infected mice. Scale bar = 25 μm. Error bars represent mean ± SEM [13].

Supporting the role of oxidative stress (OS) in the development of COVID-19 disease, we identified lipid peroxidation-derived aldehydes, namely 4-hydroxynonenal (4-HNE) and malondialdehyde (MDA), in various organs of MHV-1-inoculated mice (6 days post-MHV-1) (Figure 9A). However, treatment of MHV-1-infected mice with SPIKENET (5 mg/kg) diminished this effect (Figure 9B,C). This result offers additional evidence for the potential role of OS in the progression of SARS-CoV-2 infection.

**Figure 10:**
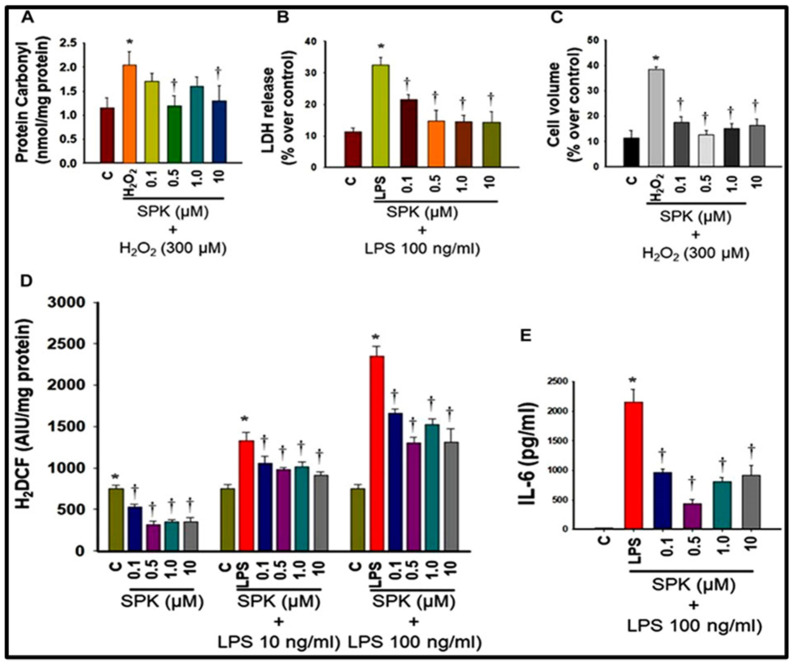
Effect of SPIKENET (SPK) on hydrogen peroxide (H2O2)-induced oxidative stress (protein carbonyl formation) (12 h) (**A**) and LPS-induced LDH release (36 h) (**B**) in primary cultures of rat brain microglia, as well as H2O2-induced increase in cell volume (24 h) in primary cultures of rat brain astrocytes (**C**). SPK significantly diminished these effects in glial cells (30 min post-treatment). Exposure of primary microglia to LPS (24 h) showed an increase in DCF fluorescence (**D**), as well as an increase in IL-6 level (**E**) in cell culture medium, and such an increase was diminished and blocked by treatment of cells (30 min post-treatment) with SPIKENET. C, control; LPS, lipopolysaccharide. * = *p* < 0.05 vs. control. † = *p* < 0.05 vs. LPS. C, control; AIU, arbitrary intensity units; LPS, lipopolysaccharide. Error bars represent mean ± SEM [13].

Another hallmark of SARS-CoV-2 infection is systemic inflammation, typically associated with generating reactive oxygen species leading to OS, which is also known to contribute to disease progression [22]. Accordingly, we also investigated SPIKENET’s impact on OS and inflammation. Our results indicate that SPIKENET attenuated a lipopolysaccharide (LPS)-induced inflammatory response and OS in primary cultures of rat brain microglia (Figure 10). Additionally, SPIKENET inhibited LPS-induced cell death (LDH release) and cellular stress in primary cultures of rat brain microglia, as well as chemically induced cell swelling in astrocyte cultures, a significant event observed in lungs post-SARS-CoV-2 infection (Figure 10).

Finally, our study also assured the safety profile of SPIKENET. Exposure to SPIKENET at high concentrations (50 and 100 μm) did not compromise cell survival or mitochondrial function in brain microglia, astrocytes, and neurons, as evaluated through the MTT assay (3-(4,5-dimethylthiazol-2-yl)-2,5-diphenyl tetrazolium bromide) while preserving its antioxidant and anti-inflammatory properties [13]. In summary, these findings further highlight the potential utility of SPIKENET in preventing SARS-CoV-2 infection and its associated long-term sequelae.

## 5. Protective Effect of SPK in Acute Water Channel Protein

**Figure 11:**
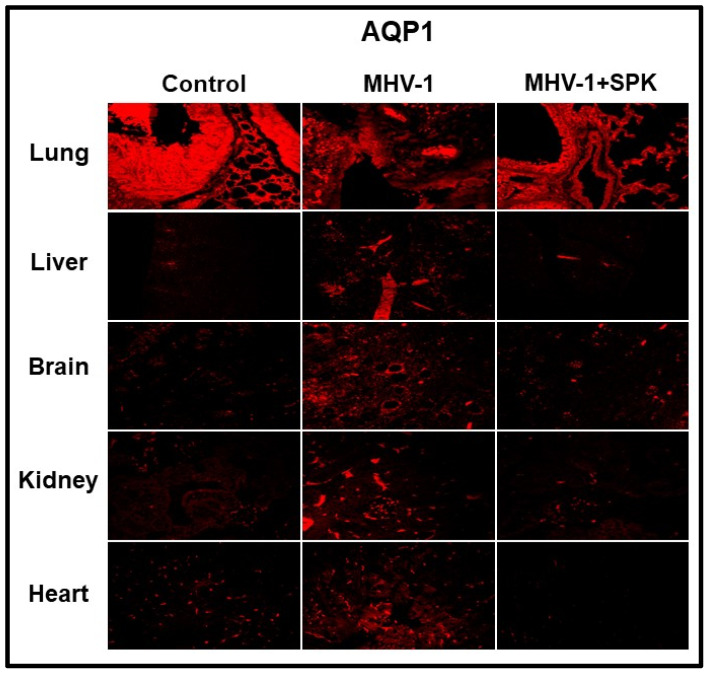
Altered AQP levels were identified in various organs post-MHV-1 inoculation. While increased AQP1 was identified in multiple organs, AQP1 levels were decreased in lungs post-MHV-1. Further, treatment of MHV-1-inoculated mice with SPK (5 mg/kg) reversed these changes. Scale bar = 25 μm [13].

Aquaporins (AQPs) are integral to maintaining intra- and extracellular fluid balance. Overexpression of aquaporins in the plasma membrane is implicated in developing edema [23,24,25,26]. We identified increases in AQPs 1, 4, 5, and 8 in every organ tissue sample analyzed in our study. Notably, AQP1 was differentially regulated (Figure 11). Treatment with SPIKENET (5 mg/kg) resulted in a reduction in AQP 4, 5, and 8 levels while returning decreased AQP1 levels to normal ranges (Figure 11) [13]. These results strongly support the central role that dysregulated AQP expression, likely secondary to oxidative stress, plays in the pathogenesis of SARS-CoV-2 infection and resultant generalized edema.

## 6. Protective Effect of SPK on Organs during Chronic Infection

**Figure 12:**
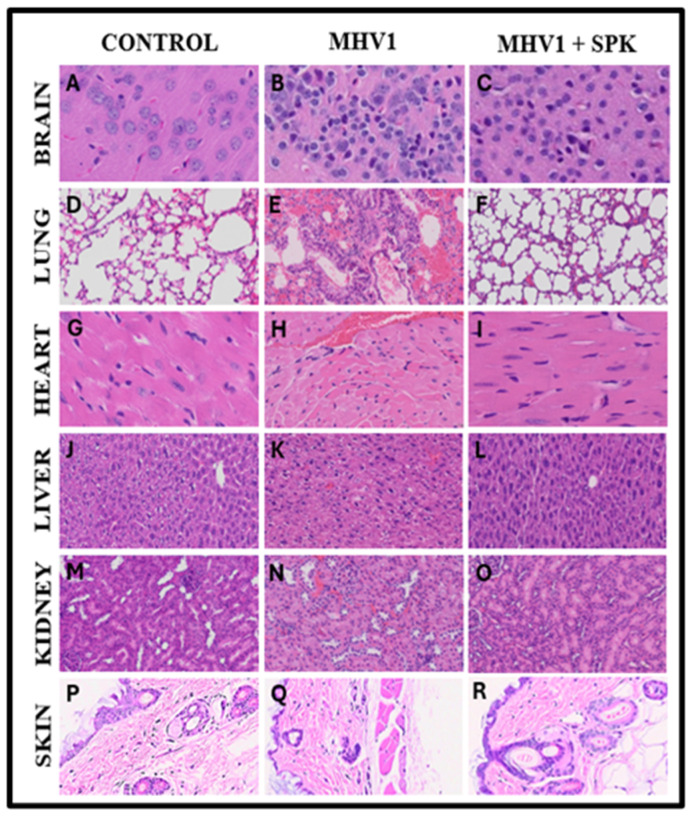
SPIKENET (SPK) diminishes MHV-1-induced pathological changes in the brain, lungs, heart, liver, kidney, and skin during long-term infection. Representative histological images of hematoxylin and eosin (H&E) stained brain, lung, heart, liver, kidney, and skin tissue sections of a standard mouse (**A**,**D**,**G**,**J**,**M**,**P**) and an infected mouse at 12 months (**B**,**E**,**H**,**K**,**N**,**Q**). MHV-1-inoculated mice treated with 5 mg/kg SPIKENET (SPK) eased all of these changes (**C**,**F**,**I**,**L**,**O**,**R**) (H&E original magnification is 400× for (**A**–**O**) and 66× for (**P**–**R**)) [8,13,21].

In the lungs of MHV-1-infected mice observed 12 months post-infection, we found severe lung inflammation, bronchiolar epithelial cell degradation, leukocyte infiltration, and hemosiderin-laden macrophages (Figure 12). Additionally, interstitial edema, thickened bronchiolar airways due to fibrotic remodeling, increased numbers of goblet cells in the epithelial lining of bronchioles, and enhanced inflammatory cell infiltration in bronchiole walls were noted [8]. Treatment with SPIKENET reversed some of these findings (Figure 12).

In the hearts of long-term COVID models, we observed severe interstitial edema, vascular congestion and dilation, and infiltration of red blood cells (RBCs) between degenerative myocardial fibers (Figure 12). Additionally, there were inflammatory cell infiltrates, apoptotic hypertrophy, and fibrosis [8]. These changes were seen during both acute and long-term conditions. SPIKENET treatment, however, effectively blunted these changes at both time points (Figure 12).

In the livers of extended COVID models of MHV-infected mice, we detected severe vascular congestion, hepatocyte degeneration, luminal thrombosis of the portal and sinusoidal vessels, hemorrhagic changes, and cell necrosis (Figure 12). Furthermore, there was increased lymphocyte infiltration in sinusoidal spaces, multifocal hepatic necrosis in periportal areas and near terminal hepatic veins, an increased number of portal veins associated with luminal severe dilation, activated Kupfer cells containing necrotic debris, eosinophilic bodies, mitotic cells, balloon-like liver cells, mild lobular lymphocytic inflammation, mild portal tract inflammation, and mild hydropic degeneration of liver parenchymal cells. Additionally, liver enzyme concentrations (ALT, ALP, AST, and Bilirubin) were significantly elevated compared to the control [8]. Again, treatment with SPIKENET diminished the extent of these pathological changes and facilitated the restoration of liver enzyme levels to normal ranges (Figure 12) [8].

## 7. Protective Effect of SPK Brain

Examination of brains from MHV-1-inoculated mice 12 months post-infection revealed widespread necrotic neurons characterized by fragmented nuclei and vacuolation. Additionally, acute injury features such as congested blood vessels, perivascular cavitation, cortical pericellular halos, vacuolation of neuropils, and darkly stained/pyknotic nuclei were observed [8] (Figure 12). These observations indicate that the long-term pathological alterations observed in the brain following infection are likely irreversible and may lead to severe neurodegeneration. Furthermore, treatment with SPK (5 mg/kg) attenuated these changes, and details of its role as a therapeutic agent are outlined below (Figure 12) [8].

Because long-term neurologic changes seen in post-infection resemble various chronic neurological conditions, we conducted a detailed examination of significant neuronal markers to assess disease severity and the therapeutic role of SPIKENET in preventing neurologic pathology. Our analysis included assessing hyperphosphorylated TDP-43 and tau, the NR1 subunit of the NMDA receptor, astrocytic and microglial activation, and the presynaptic protein synaptophysin-1 [8]. We found increased reactive astrocytes and microglia in the cerebral cortex of MHV-1-infected mice, which were reduced following treatment with 5 mg/kg SPK. Similarly, elevated levels of phosphorylated TDP-43 and tau were observed in these mice. At the same time, those who underwent treatment with SPK (5 mg/kg) exhibited comparatively lower levels suggestive of SPK’s protective effects. Furthermore, an almost complete loss of synaptophysin-1 was observed in MHV-1-infected mice, but this effect was significantly reduced with the treatment of SPK (5 mg/kg) [8]. These results further highlight the significant risk for long-term neurologic pathology in COVID-19 disease as well as the critical role of SPIKENET as an effective treatment.

## 8. Protective Effect of SPK in Renal Fibrosis

**Figure 13:**
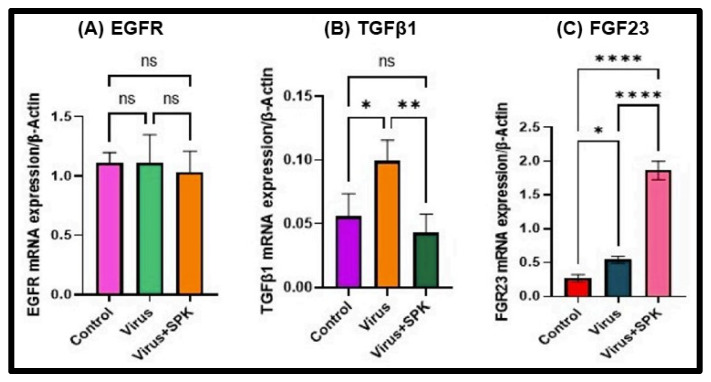
Altered mRNA expressions 12 months post-MHV-1 infection (chronic). (**A**) EGFR mRNA level does not significantly change in disease and treatment with SPK. (**B**) TGF-β1 mRNA level is significantly increased in the infected group, while its mRNA level is decreased substantially following SPK treatment. (**C**) FGF-23 mRNA level is significantly increased in the infected group, and therapy with SPK leads to substantial elevation. Values are the mean SD of three independent experiments. * = *p* < 0.05, ** = *p* < 0.01, and **** = *p* < 0.0001 are statistically significant. ns = nonsignificant [20].

Growth factors and their associated cellular signaling pathways have been implicated in the pathogenesis and progression of fibrosis in various organs [27,28,29,30]. Pulmonary fibrosis is seen in individuals infected with SARS-CoV 2 and is believed to be mediated by the epidermal growth factor receptor (EGFR) signaling pathway [27,28]. Our earlier histopathological analyses of the kidney illustrated severe and irreversible kidney dysfunction in both short and long-term post-infection periods [20]. While EGFR stimulation is associated with renal fibrosis in other clinical conditions [29], its role during acute and long-term SARS-CoV-2 infection is not fully understood. We employed our surrogate mouse model [20] to determine the extent to which EGFR stimulation is responsible for renal fibrosis in COVID-19 disease. We determined that EGFR levels exhibited no significant alterations in long COVID models regardless of treatment with SPIKENET (Figure 13) [20].

Transforming growth factor-beta (TGF-β) is widely recognized as a critical contributor to fibrosis development and progression in various organs. Its involvement primarily entails the activation of canonical and non-canonical signaling pathways, resulting in the deposition of extracellular matrix (ECM) [29,30]. We found a significant increase in TGF-β mRNA levels in kidneys 12 months post-MHV-1 infection (Figure 13) [20], supporting its involvement in developing renal fibrosis during cases of long COVID. On the other hand, TGF-β mRNA levels in MHV-1-infected mice treated with SPIKENET were significantly reduced (Figure 12), offering additional evidence for the integral role TGF-β plays in renal fibrosis secondary to SARS-CoV-2 infection [20].

Another growth factor, FGF23, is known to rise in response to inflammation and hypoxia and can contribute to the development of chronic kidney disease (CKD) [31]. Notably, elevated FGF23 levels have been documented in COVID-19-infected patients with a history of CKD [32]. In the long-term post-infection group, we observed a significant increase in FGF23 expression, further exacerbated by treatment with SPK (5 mg/kg) (Figure 13) [20]. While the reason for such an effect of SPK on FGF23 is unclear, we speculate that SPK may have increased the FGF23 with mechanisms independent of inflammatory stimulation.

## 9. Protective Effect of SPK in Skin

Remarkably, treating MHV-1-infected mice with the synthetic peptide SPK substantially moderated cutaneous changes associated with long COVID [21]. SPK intervention effectively restored hair follicle morphogenesis (Figure 12), in stark contrast with the untreated control group (Figure 12). Intriguingly, this therapeutic intervention reinstated architectural integrity in adipose tissue and across dermal and epidermal layers, including sebaceous glands (Figure 12), compared to the control group (Figure 12). Additionally, sebaceous glands exhibited notable dispersion along hair follicle lengths, accompanied by discernible thickening of the panniculus carnosus (Figure 12), notably absent in untreated controls (Figure 12). In Figure 12Q, diverse stages of hair follicle degeneration are evident long-term post-infection, juxtaposed with standard, healthy mice, as illustrated in Figure 12. The application of SPK treatment led to the restoration of hair follicles across distinct stages, coupled with adipose tissue regeneration. SPK treatment also reversed cutaneous abnormalities observed in chronic infection, restored hair follicle numbers, and reorganized the architecture of epidermal and dermal layers, repairing adipose tissue [21]. As observed previously in the kidney, SPK administration reduced TGF-β levels. TGF-β initiates an intercellular signaling cascade that stimulates fibroblasts responsible for collagen production deposited in the ECM. This suggests SPK can mitigate collagen and fibrosis accumulation in the ECM by regulating TGF-β [21].

**Figure 14:**
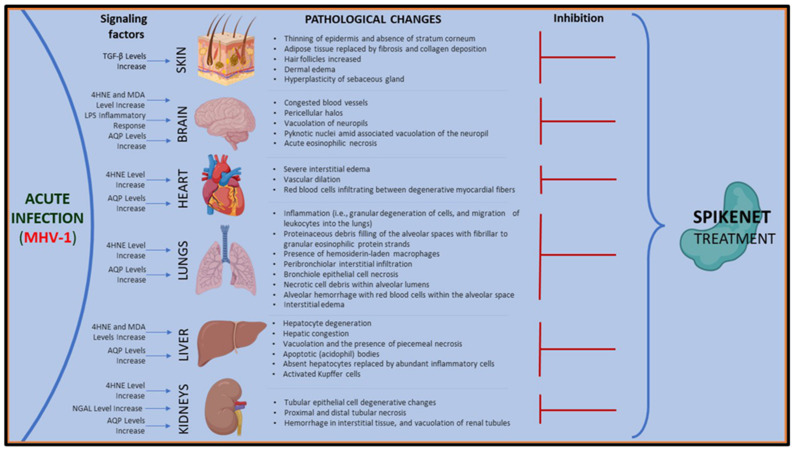
The MHV-1 coronavirus-mediated signaling systems in various organs result in pathological and functional consequences. SPK ameliorated or prevented such defects in these organs.

**Figure 15:**
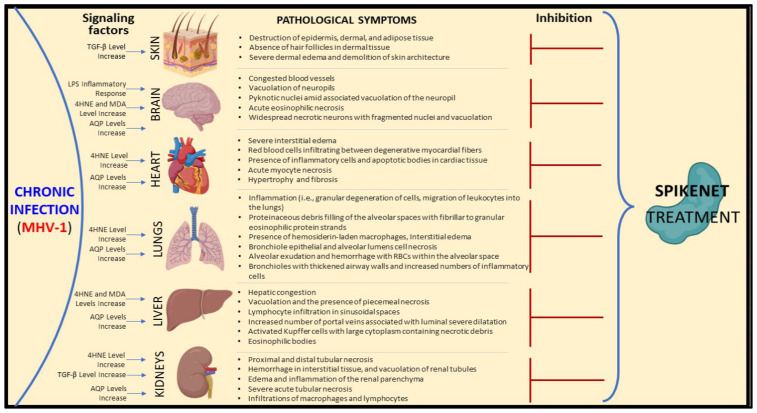
MHV-1 coronavirus instigated signaling pathways across diverse organs, leading to pathological and functional repercussions. SPK mitigated or averted such abnormalities within these organs.

Our investigations increasingly demonstrate that administering SPK effectively prevents both acute and long-term complications of COVID-19 (Figure 14 and Figure 15). These findings suggest SPK addresses immediate symptoms and complications associated with acute COVID-19 infection and is crucial in mitigating the risk of long-term health issues post-infection. This discovery holds significant promise for improving public health outcomes and reducing the burden on healthcare systems worldwide. Furthermore, the efficacy of SPK in preventing COVID-19 complications underscores the importance of continued research and development efforts in identifying effective therapeutic interventions against emerging infectious diseases.

## 10. Side Effects

Though generally considered safe, protein-based peptides can elicit various side effects contingent on factors like their structure, dosage, and administration route. Allergic reactions, ranging from mild itching to severe anaphylaxis, can occur, especially in individuals sensitive to similar protein sources [33]. Injection-site reactions, such as pain or swelling, may manifest with injectable peptide drugs. Gastrointestinal disturbances like nausea or diarrhea may arise from orally administered peptides. Immune system modulation with peptides may lead to immunogenicity or autoimmune reactions [33]. Metabolic, cardiovascular, endocrine, and central nervous system effects are also possible, depending on the peptide’s target pathway.

Outbreaks such as severe acute respiratory syndrome (SARS) in 2002, influenza A/H1N1 in 2009, Ebola in 2013, and SARS-CoV-2 in 2019 underscore the persistent threat of viral pathogens, owing to their proclivity for mutation. Consequently, the imperative to devise antiviral strategies remains paramount. Enzyme inhibitors, given the pivotal roles of enzymes in viral replication and the established methodologies for their discovery, constitute a primary focus. However, the exigencies of viral evolution necessitate a multifaceted approach, including exploring protein–protein interaction (PPI) inhibitors. PPIs represent promising therapeutic targets not only in cancer but also in combating viral infections, as demonstrated by the approval of Enfuvirtide in 2003 for HIV treatments and ongoing efforts targeting various viruses such as HCV and SARS-CoV-2. Notably, the interaction between viral proteins presents a promising avenue for intervention. Three primary avenues—low-molecular-weight compounds, peptide-based inhibitors, and antibodies—stand out for their potential to inhibit viral PPIs, although each has distinct advantages and disadvantages. While small molecules face hurdles due to their low binding surface, medium-sized compounds like peptides offer spatial flexibility. In essence, this discourse delineates diverse strategies for developing antiviral PPI inhibitors and synthetic peptides encompassing the identification of molecular targets and the exploration of compounds across different classes, underscoring the dynamic landscape of antiviral therapeutics [34,35].

## 11. Pharmacodynamic

The pharmacodynamics of protein-based peptides involves their interaction with the body’s receptors and signaling pathways to produce therapeutic effects [36,37]. These peptides, composed of short chains of amino acids, exert diverse biological activities such as hormone regulation, neurotransmission modulation, and immune system modulation. They typically bind to specific receptors on cell surfaces, initiating signal transduction pathways within cells. This binding triggers downstream events like enzyme activation or inhibition, modulation of gene expression, immune modulation, and metabolic effects [36]. Protein-based peptides can act as agonists or antagonists, stimulating or inhibiting physiological responses depending on their target and mechanism of action [37]. Understanding the pharmacodynamics of protein-based peptides is crucial for optimizing their therapeutic efficacy and developing novel treatments for various medical conditions. This comprehension requires elucidating the molecular mechanisms underlying their interactions with biological targets and their effects on cellular and physiological processes [36,37].

## 12. Pharmacokinetic

Following the administration of protein-based therapy, it enters circulation and is distributed throughout the body, often targeting specific tissues or cells [37,38]. The metabolism of protein-based therapies primarily occurs via enzymatic breakdown in the blood and tissues, leading to the generation of smaller peptides and amino acids that are eliminated through renal clearance or hepatic degradation [38]. Pharmacokinetic parameters such as clearance, volume of distribution, and half-life play crucial roles in determining dosing regimens and optimizing therapeutic efficacy.

## 13. Conclusions

In summary, SPIKENET (SPK) shows significant potential as a treatment for diminishing and alleviating symptoms of acute and long COVID-19 in humans, as demonstrated in the MHV1 mice model. The mechanism of SPK binding to SARS-CoV-2 spike glycoprotein-1 (S1), preventing S1 from binding to the human ACE2 receptor, is implied to inhibit viral entry into host cells. Additionally, SPK mitigated abnormal protein expression in many organs, attenuating their acute and long-term COVID symptoms, such as edema, renal fibrosis, and tissue necrosis. However, further studies are warranted to observe further the effects of SPIKENET on other vital organs and its efficacy on other COVID models to underscore its applicability as a chronic COVID treatment.

## Data Availability

The data presented in this study are available upon request from the corresponding author. However, due to the University of Miami Miller School of Medicine’s privacy policy, they are not publicly available.
